# A new Australian species of *Luffa* (Cucurbitaceae) and typification of two Australian *Cucumis* names, all based on specimens collected by Ferdinand Mueller in 1856

**DOI:** 10.3897/phytokeys.5.1395

**Published:** 2011-07-27

**Authors:** Ian R. H. Telford, Hanno Schaefer, Werner Greuter, Susanne S. Renner

**Affiliations:** 1 School of Environmental and Rural Science, University of New England, Armidale, NSW, 2351, Australia; 2 Organismic and Evolutionary Biology, Harvard University, 22 Divinity Avenue, Cambridge, MA-02138, USA 20013-7012; 3 Herbarium Mediterraneum, c/o Orto Botanico, Via Lincoln 2/A, I-90123 Palermo, Italy; 4 Systematic Botany and Mycology, University of Munich, Menzinger Strasse 67, 80638, Munich, Germany 20013-7012

**Keywords:** Ferdinand Mueller, melon, wild relatives, *Cucumis picrocarpus*, *Cucumis jucundus*, *Luffa saccata*, lectotypification

## Abstract

As a result of his botanical explorations in northern Australia, Ferdinand von Mueller named several Cucurbitaceae that molecular data now show to be distinct, requiring their resurrection from unjustified synonymy. We here describe and illustrate *Luffa saccata* F. Muell. ex I.Telford, validating a manuscript name listed under *Luffa graveolens* Roxb. since 1859, and we lectotypify *Cucumis picrocarpus* F. Muell. and *Cucumis jucundus* F. Muell. The lectotype of the name *Cucumis jucundus*, a synonym of *Cucumis melo*, is mounted on the same sheet as the lectotype of *Cucumis picrocarpus*, which is the sister species of the cultivated *Cucumis melo* as shown in a recent publication.

## Introduction

Ferdinand von Mueller (1825–1896) was the botanist on the North Australian Exploring Expedition that in 1855 and 1856 explored North Australia under the command of A. C. Gregory (Gregory 1858; maps of the expedition are available at http://nla.gov.au/nla.map-rm2807). In mid-September 1855, the expedition’s two ships reached the mouth of the Victoria River, and the explorers then spent eight months exploring the surrounding country. They started their return journey on 21 June 1856. Mueller is not known to have visited the Northern Territory again later (see Orchard 1999, and literature cited there), and although many of his specimens are undated they can be associated with confidence with the North Australian Exploring Expedition on the basis of the locality data.

Many new taxa were collected on that expedition, including two new species of melon described as *Cucumis jucundus* F. Muell. and *Cucumis picrocarpus* F. Muell. (Mueller 1859). Both names were subsumed into *Cucumis trigonus* Roxb. by Bentham (1866), who however noted that they might be forms of *Cucumis melo* L. In 1993 (pp. 104 and 114), Kirkbride included both as synonyms under *Cucumis melo* without assigning them to a definite infraspecific taxon. Some of the specimens in CANB and MEL thus referred to *Cucumis melo* had earlier (in 1986) been annotated by Charles Jeffrey as *Cucumis melo* subsp. nov., and Kirkbride (1993) also commented on the Australian material’s polymorphism in the degree of leaf dissection and the indumentum of the hypanthium of female flowers.

A phylogenetic reconstruction of *Cucumis* that includes over 100 accessions from Asia and Australia now indicates that some of the Australian material previously referred to *Cucumis melo* constitutes distinct species (Sebastian et al. 2010). The molecular data show that Australia harbors seven native species of *Cucumis*, five of them new to science and described elsewhere (Telford et al. 2011). Examination of Mueller’s collections and protologues indicates that Mueller’s name *Cucumis picrocarpus* applies to the Australian sister species of the worldwide crop *Cucumis melo* (and its wild progenitor forms native in India), while Mueller’s *Cucumis jucundus* is a synonym of *Cucumis melo*. The possible importance to plant breeders of this Australian sister to *Cucumis melo* makes it expedient to designate the types of Mueller’s Australian *Cucumis* names, a task carried out here.

Ongoing molecular-phylogenetic work on *Luffa* vindicates another of Mueller’s suspected new species, this one never formally described by him. Like the two melon species, he discovered it in the Victoria River region, and there are at least two specimens labeled by Mueller as ‘*Luffa saccata*.’ Mueller’s manuscript name was listed as a synonym under *Luffa graveolens* Roxb. by Naudin (1859), a famed Cucurbitaceae specialist, with the result that Mueller’s name went unnoticed for the next 150 years. *Luffa graveolens* occurs in India, Nepal, Bangladesh, and Burma, and is morphologically distinct from the Australian species. We here validate the name *Luffa saccata*, describe the morphological differences between *Luffa graveolens* and *Luffa saccata*, and provide illustrations.

## Taxonomic results and discussion

### Emended lectotypification of Cucumis jucundus:

Cucumis jucundus F. Muell., *Trans. Philos. Inst. Victoria* 3: 45 (1859) as ‘jucunda’.

Lectotype (designated here): AUSTRALIA. Northern Territory: Victoria River, undated, *F. Mueller* (K000634446!, p.p., excluding upper stem with fruit attached; isolectotypes: Same locality, undated, GH000312219! [as *‘Cucurbita jucunda’*], K000634445!, MEL000592946! [as *‘Cucurbita jucunda’*]). A second sheet at GH from “Depot Creek” and one at MEL000592947 from “Victoria River, Depot Creek” are possible further isolectotypes.

**Figure 1.**
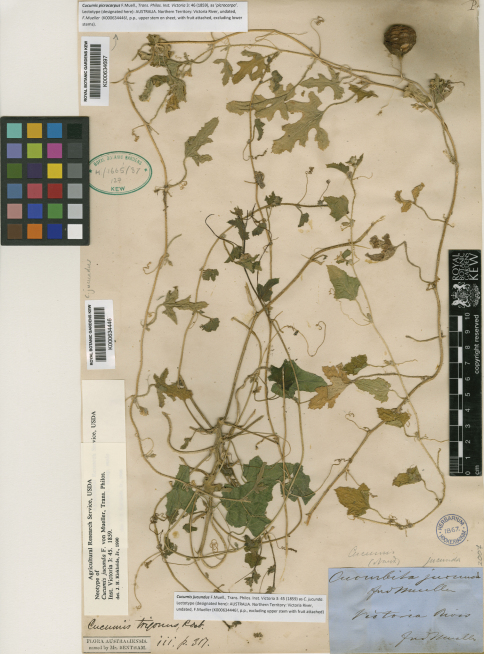
The Kew sheet K000634697 and K000634446 with the mixed collection of two species of *Cucumis* collected by Ferdinand von Mueller in Australia. The stem with the deeply lobed yellowish leaves and the attached fruit is the lectotype of *Cucumis picrocarpus* F. Muell., while the branch with the more green and much less lobed leaves is the lectotype of *Cucumis jucundus* F. Muell.

Kirkbride (1993) designated a sheet in the Kew Herbarium (now K000634446; our Fig. 1) as ‘neotype’ of *Cucumis jucundus* and another one (now K000634445) as ‘neoisotype’ [sic]. In fact, the material on K000634446 represents two species, *Cucumis jucundus* and *Cucumis picrocarpus*, both discovered by Mueller during the North Australian Exploring Expedition and described by him (Mueller 1859) on the basis of his own collections. Kirkbride’s ‘neotype’ thus comprises original material for both *Cucumis jucundus* and *Cucumis picrocarpus*. Under the Vienna Code (McNeill et al. 2006: Art. 9.8), the term neotype is, in such cases, correctable to lectotype. However, the sheet referred to by Kirkbride is not a specimen as defined in the Code (Art. 8.2), because it does not consist of ‘a single species’. A “second stage lectotypification” as provided for in Art. 9.12 is therefore necessary, to ensure that the name *Cucumis jucundus* F. Muell. remains attached to those plant parts on K000634446 that “correspond most nearly with the original description or diagnosis.” At least five other sheets annotated by Mueller as *Cucumis jucundus* or *Cucurbita jucunda* and coming from different collecting localities (without collecting dates) are kept at MEL, the herbarium of Mueller’s home institution, and the protologue statement about the geographic range of *Cucumis jucundus* is accordingly broad: ‘In Arnhem’s Land and on the Gulf of Carpentaria, particularly on the banks of rivers, also in eastern tropical Australia, and in Central Australia observed with certainty as far south as Cooper’s River’ (Mueller 1859: 45). The sheet MEL000592946, with male flowers, was collected at ‘Victoria River’ and is undoubtedly a duplicate of our lectotype, same as one of the two specimens kept at GH. A second sheet at GH from “Depot Creek” and one at MEL from “Victoria River, Depot Creek” are possible further isolectotypes.

### Lectotypification of Cucumis picrocarpus:

Cucumis picrocarpus F. Muell., *Trans. Philos. Inst. Victoria* 3: 46 (1859), as ‘*picrocarpa’*.

Lectotype (designated here): AUSTRALIA. Northern Territory: Victoria River, undated, *F. Mueller* (K000634697!, p.p., upper stem on sheet, with fruit attached, excluding lower stems).

The upper stem on the sheet K000634697 (our Fig. 1), with an attached fruit, is the only extant specimen of *Cucumis picrocarpus* known to have been collected prior to publication of the name. Neither a specimen annotated as *Cucumis ‘picrocarpa’* by Mueller nor any other original material has been located in MEL. There is thus no option than designating K000634697 as lectotype. The specimen exhibits the deeply lobed leaves and fruit indumentum described in Mueller’s protologue, which does not cite a specimen, only a distributional statement: ‘In many parts of tropical Australia’ (Mueller 1859: 46). According to label information on K000634697, Mueller’s plant was collected in the Victoria River region, where *Cucumis picrocarpus* grows sympatrically with feral forms of *Cucumis melo*, also collected there by Mueller and annotated by him as *Cucurbita jucunda* or *Cucumis jucundus* (compare our first lectotypification, above). An emended description of *Cucumis picrocarpus*, a distribution map, and an illustration are provided in Telford et al. (2011).

### Description of Luffa saccata:

#### Luffa saccata

F. Muell. ex I. Telford sp. nov.

urn:lsid:ipni.org:names:77112771-1

http://species-id.net/wiki/Luffa_saccata

##### Holotype.

AUSTRALIA. Northern Territory: Baines Creek [=Baines River, a tributary of the Victoria River], May 1856, *F. Mueller* (MEL000593093!, the fragment pocket contains three seeds; isotypes: K000634638, K000634639, K000634640, the latter with a tag in Mueller’s handwriting ‘*Luffa saccata* Ferd. Mueller. Tropical Australia. Victoria River. May 1856’).

**Figure 2.**
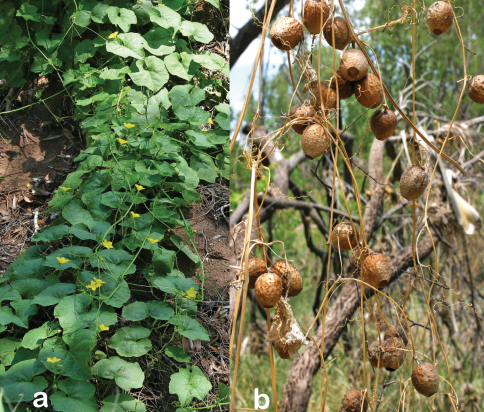
**a** Habit of *Luffa saccata* F.Muell. ex I.Telford **b** Old fruits. Photos taken near the type locality, Gregory National Park, Northern Territory, by A. Rodd.

**Figure 3.**
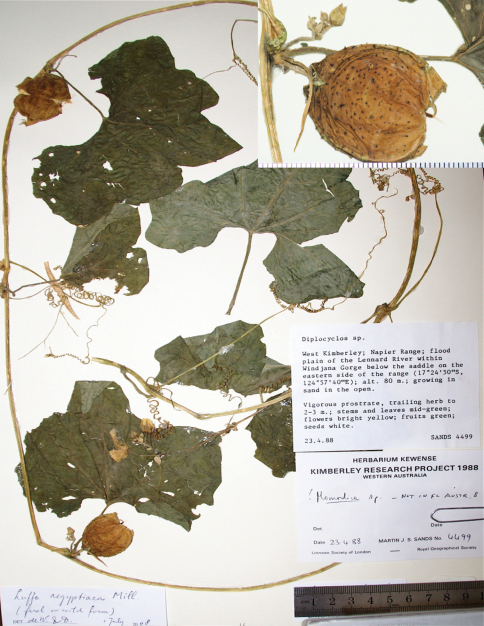
Typical herbarium specimens of *Luffa saccata*: *Sands 4499* (L).

A *Luffa graveolente* floribus masculis longe pedicellatis in racemo elongato dispositis (nec brevissime pedicellatis subfasciculatis) et pedicellis fructiferis quam 15 mm brevioribus (nec plusquam 15 mm longis) differt. Our Figs. 2 and 3.

Trailing or climbing annual herb, monoecious; stems to 7 m long, 2–3 mm diam., ± glabrous, ribbed. Tendrils 3–5-branched, the stem 4.5–8 cm long, branches 5–9 cm long. Leaves: petiole 1.5–6.5 cm long; lamina ovate to broadly ovate in outline, 3–14 cm long, 2.5–13 cm wide, with 3 or 5 broad rounded or obtuse lobes, the lobes crenate, base cordate with the sinus wide, apex acute, hispid on both surfaces. Inflorescences usually unisexual, rarely with male and female flowers. Male flowers in elongate racemes, rarely solitary; racemes 10–16-flowered, 3–10(–30) cm long; peduncles 1.5–12 cm long; bracts ovate, 2–3 mm long, glandular; pedicels of racemose inflorescences 5–20 mm long, of solitary flowers 8–64 mm long; hypanthium broadly campanulate, 2–3 mm long; calyx lobes 5, triangular, 4–10 mm long, puberulous abaxially; corolla lobes 5, ovate–broadly spathulate, 12–20 mm long, ± glabrous, yellow; stamens 3, inserted towards the base of the hypanthium; anthers one 1-thecous, two 2-thecous, flexuose; disc absent. Female flowers: solitary, sometimes paired in axils; pedicels 3–15(–20) mm long; ovary ovoid, 8–12 mm long, long-attenuate, pilose, shortly echinate; hypanthium above the constriction and perianth similar to male; staminodes 3; style c. 3 mm long; stigmas 3, 2-lobed, c. 2 mm long. Fruit ovoid, 2.5–4.5 cm long, 2–4 cm diam., glabrescent, echinate, many-seeded, dehiscing by an apical operculum; fruiting pedicel 2–15(–20) mm long. Seeds elliptic, 7–8 mm long, 4–5 mm wide, smooth or slightly rugose, dark brown mottled black, the margin narrowly winged.

##### Representative specimens examined.

AUSTRALIA. Western Australia: Fitzroy River floodplain, river road from Minnie River bridge to Udialla homestead, 27 Apr. 1993, *A.A. Mitchell 3040* (CANB); Geikie Gorge, mouth of gorge, 14 May, 1992, *I.R. Telford 11721* (CANB); Napier Range, flood plain of Lennard River within Windjana Gorge, 23 Apr. 1988, *M.J.S. Sands 4499* (K, L, PERTH); Napier Range, Tunnel Creek, 8 Apr. 1988, *C.R. Dunlop 7757* (BRI, DNA); c. 2 km SW of Crystal Heads, *A.A. Mitchell 3352* (CANB, PERTH); Mitchell River Falls, Mitchell Plateau, 22 Jan. 1982, *K.F. Kenneally 7896* (BRI, PERTH); Lower Ord River, 4 km W of Tarara Bar, 6 July 1994, *K.F. Kenneally 11519* (CANB, PERTH); Ord River, *C.R. Dunlop*, seeds cultivated at Bloomington University, *C.B. Heiser 1979* (IU). Northern Territory: Victoria River, 12 km W of Timber Creek, 14 Jul. 1977, *J. Must 1630* (BRI, CANB, DNA, NT); Lejeune Station, Barramundi Dam, 21 Feb. 1994, *G.J. Leach 4086* (BRI, DNA); Wickham River, *J. Russell-Smith 7752 & D.E. Lucas* (BRI, CANB, DNA); McArthur River area, sandstone plateau above Glyde River, 17 Feb. 1977, *L.A. Craven 3898* (CANB, DNA).

##### Distribution.

Widespread in the Kimberley, Western Australia and the adjacent north-western Northern Territory, with a disjunction to the McArthur River area of the Northern Territory. Australia’s Virtual Herbarium (http://avh.rbg.vic.gov.au/avh/ accessed 23 March 2011) provides locations for some 50 collections in Australian herbaria, still under the name *Luffa graveolens*.

##### Habitat.

*Luffa saccata* grows in riverine or littoral habitats on sand or clay, sometimes on rocky ridges of limestone or sandstone to 300 m of altitude. Associated species recorded include *Eucalyptus camaldulensis*, *Melaleuca leucadendra* and *Barringtonia acutangula*  in gallery forest or woodland, and *Eucalyptus miniata*, *Adansonia gregorii*, *Brachychiton* spp. and *Triodia* spp. on ridges and littoral *Cenchrus* grassland.

##### Phenology.

Flowers and fruits March to October.

##### Conservation Status.

The species is widespread and common, and we therefore do not consider it at risk. Conserved in Mitchell River and Bungle Bungle Ntional Parks in Western Australia and Gregory National Park in the Northern Territory.

##### Etymology.

From Latin *saccatus,* bag-like, obviously in reference to the fruit (Figs 2, 3).

##### Notes.

The MEL holotype has two labels in Mueller’s handwriting, one with ‘*Luffa saccata* Baines Creek, May 1856’, the other with ‘*Luffa graveolens*, Tributaries of the Victoria River, N.W. Australia, May 1856,’ the latter obviously attached after communication with, or reading of, Naudin (1859). It is surprising that Naudin failed to accept Mueller’s Australian *Luffa* as a good species, since C.B. Clarke (1832–1906), who knew the Indian cucurbits well, made a note on one of the three Kew specimen, saying ‘not near [*Luffa*] *graveolens* which has the males [male flowers] on very short subfasciculate pedicels.’ This is indeed one of the differences between the Indian and the Australian species, the latter having the male flowers mostly in elongate racemes. Detailed measurements of living Indian *Luffa graveolens* plants, black and white photos, and observations on their chromosome numbers are contained in (Dutt and Roy (1969, 1971).

No material of *Luffa graveolens* from India is held in the following major herbaria: CGE, E, GH, L, MO, NY, US. This lack of material in western herbaria probably contributed to the Indian and Australian species having been confused for so long. The confusion also affected a recent treatment of Cucurbitaceae in the *Flora Malesiana* series (De Wilde and Duyfjes 2010), which states that *Luffa aegyptiaca* forma *sylvestris* (Miq.) W.J.de Wilde & Duyfjes is common in Australia (and elsewhere) and comprises “all wild-growing and naturalized small-fruited feral forms” of *Luffa*. Several Australian specimens of *Luffa saccata*, such as *Sands* 4499 (Fig. 3), thus are annotated as *Luffa aegyptiaca* forma *sylvestris*. The *Luffa* specialist C.B. Heiser, on the other hand, cultivated both Australian species, *Luffa aegyptiaca* and *Luffa saccata* (under the name *Luffa graveolens*), and distinguished them without hesitation (Heiser and Schilling 1988).

## Supplementary Material

XML Treatment for Luffa saccata
